# Modulation of airway epithelial cell functions by Pidotimod: NF-kB cytoplasmatic expression and its nuclear translocation are associated with an increased TLR-2 expression

**DOI:** 10.1186/1824-7288-39-29

**Published:** 2013-05-10

**Authors:** Sonia Carta, Michela Silvestri, Giovanni A Rossi

**Affiliations:** 1 Pediatric Allergy and Pulmonary Disease Unit, Istituto Giannina Gaslini, Via G Gaslini 5, Genoa, 16147, Italy

**Keywords:** Recurrent respiratory infections, Immunostimulants, Toll like receptor-2, ICAM-1

## Abstract

**Background:**

Recurrent respiratory infections are one of the most important causes of morbidity in childhood. When immune functions are still largely immature, the airway epithelium plays a primary defensive role since, besides providing a physical barrier, it is also involved in the innate and the adaptive immune responses. A study was therefore designed to evaluate in vitro whether pidotimod, a synthetic dipeptide able to stimulate the inflammatory and immune effector cells, could activate bronchial epithelial cell functions involved in response to infections.

**Methods:**

BEAS-2B cell line (human bronchial epithelial cells infected with a replication-defective Adenovirus 12-SV40 virus hybrid) were cultured in the presence of pidotimod, with or without tumor necrosis factor (TNF)-α or zymosan to assess: a) intercellular adhesion molecule (ICAM)-1 expression, by flow cytometry; b) toll-like receptor (TLR)-2 expression and production, by immunofluorescence flow cytometry and western blotting; d) interleukin (IL)-8 release, by enzyme-linked immunosorbent assay (ELISA); e) activated extracellular-signal-regulated kinase (ERK1/2) phosphorylation and nuclear factor-kappa B (NF-kB) activation, by western blotting.

**Results:**

The constitutive expression of ICAM-1 and IL-8 release were significant up-regulated by TNF-α (ICAM-1) and by TNF-α and zymosan (IL-8), but not by pidotimod. In contrast, an increased TLR-2 expression was found after exposure to pidotimod 10 and 100 μg/ml (p < 0.05) and to the association pidotimod 100 μg/ml + TNF-α (p < 0.05). Western blot analysis substantiated that the constitutive TLR-2 expression was significantly increased after exposure to all the stimuli. Finally, while a remarkable inhibition of TNF-α -induced ERK1/2 phosphorylation was observed in the presence of pidotimod, both TNF-α and pidotimod were effective in inducing NF-kB protein expression in the cytoplasm and its nuclear translocation.

**Conclusion:**

Through different effects on ERK1/2 and NF-kB, pidotimod was able to increase the expression of TLR-2 proteins, surface molecules involved in the initiation of the innate response to infectious stimuli. The lack of effect on ICAM-1 expression, the receptor for rhinovirus, and on IL-8 release, the potent chemotactic factor for neutrophils (that are already present in sites of infection), may represent protective functions. If confirmed in vivo, these activities may, at least in part, clarify the mechanism of action of this molecule at airway level.

## Background

Respiratory tract infections, presenting as common cold, rhinosinusitis, tonsillopharyngitis, otitis media and tracheo-bronchitis, with or without airway obstruction, are highly prevalent among young children [1-3]. These infections have not only an impact on children’s health and well-being, but also generate high medical costs and indirect costs for the family and the society [4,5]. Indeed, on average, young children experience 4–6 upper respiratory tract infections per year [6], but when they grow older, the incidence of these infections decreases, probably as a result of a more mature immune defenses and improved anatomical conditions. The most common pathogens involved in the etiology of recurrent respiratory infections are human rhinoviruses (HRV), adenovirus, parainfluenza virus, respiratory syncytial virus, enterovirus, human metapneumovirus and coronavirus, in addition to influenza viruses and rhinovirus [6,7]. A major pathogenetic role is played by rhinoviruses, the most frequent causative agents of both upper and lower respiratory tract infections in infants and young children that are able to induce a broad variety of clinical outcomes, ranging from asymptomatic infections to severe respiratory diseases requiring hospitalization [6,8].

Being protected by a highly specialized innate and adaptative immune system, the surface of the respiratory tract acts as a selective barrier, maintaining the integrity of tissue compartments and impeding entry of inhaled majority microbial pathogens, irritants and allergens [9]. Besides providing a physical and functional barrier to external agents, airway epithelial cells are also actively involved in initiation of the host inflammatory and immune responses through the release of early inflammatory mediators [9,10]. Through the activation of their surface pattern recognition receptors, that detect environmental stimuli, airway epithelial cells secrete endogenous danger signals, thereby activating dendritic cells and bridging innate and adaptive immunity [9-11].

Extensive research into the role of inflammatory mediators in the pathogenesis of respiratory insufficiency syndrome (RIS), has produced evidence for increased concentrations of several mediators, such as kinins, leukotrienes, histamine, interleukins 1, 6, and 8, tumor necrosis factor (TNF)-α, and regulated by activation normal T cell expressed and secreted (RANTES) in the nasal secretions of patients with colds [12-14]. The host response mechanisms triggered by viral infection and their efficacy in protecting the host are, however, extremely complex and far from being resolved. Furthermore an exaggerated inflammatory reaction, may increase the damage at airway levels, rather than protect from infection [12-15].

Several studies have shown that atopy and attendance at large daycare are associated with more common respiratory infections during the preschool years [16,17], while only in a minority of patients partial IgA and/or IgG subclass deficiency can be demonstrated [18]. However, a defective (immature) inflammatory and/or immune response at airway epithelial level may also be involved [19].

With this background a study was designed to evaluate in vitro whether pidotimod, a synthetic dipeptide active on both innate and adaptive immunity [20], could modulate airway epithelial cells functions involved in the response to respiratory infections. We evaluate intercellular adhesion molecule (ICAM)-1 and toll like receptor (TLR)-2 expression, interleukin (IL)-8 release and investigate the possible involvement of the protein complex nuclear factor kappa-light-chain-enhancer of activated B cells (NF-kB) and the activated extracellular-signal-regulated kinase (ERK)1/2 phosphorylation.

## Materials and methods

### Cell culture

A human bronchial epithelial cells line (BEAS-2B) (ATCC, Manassas, VA, USA), derived from human bronchial epithelium transformed by an adenovirus (12-SV40 hybrid virus) was used in all the experiments [21]. These cells, that retain electron microscopic features of epithelial cells and show positive staining with antibodies to cytokeratin, were grown as monolayer in a 1:1 mixture of Laboratory of Human Carcinogenesis (or LHC)-9 medium (Invitrogen SRL; Milan, Italy) and RPMI 1640 medium (EuroClone; Milan, Italy).

### Pidotimod preparation

Pidotimod (99.6% purity) was gently provided by Valeas S.p.A. (Milan, Italy). Stock standard solution was prepared in PBS at the concentration of 35 mg/ml [20].

### Assessment of cell viability

BEAS-2B were treated with pidotimod (10, 100 μg/ml), 10 ng/ml TNF-α or 50 μg/ml zymosan used alone or in association, at different time (24 and 48 h), the percent viability was measured by trypan blue dye exclusion test (EuroClone S.p.A., Mi, Italy) [22]. Cells were counted in a Neubauer chamber and viable cells were detected based on the ability to exclude the dye. Non-viable cells were blue due to defects in the cell membrane.

### Flow cytometry

BEAS-2B cells were grown to 90% confluence in 12 well culture plate in the presence of pidotimod (10 and 100 μg/ml), 10 ng/ml TNF-α, 50 μg/ml zymosan used alone or in association, or pre-treated with pidotimod for different time and then stimulated with TNF-α or zymosan for another 24 h. For each analysis 2 × 10^6^ cells were incubated with FITC-Conjugated mouse anti ICAM-1 (Invitrogen S.r.l., San Giuliano Milanese, Italy) and with an anti-mouse TLR-2 (Santa Cruz Biotechnology Inc, Segrate, Milan, Italy) antibody for 1 h [22]. TLR-2 expression was detected using a goat anti-mouse antibody (Alexa Fluor488; Invitrogen). The cells were washed, resuspended in PBS and immediately analyzed with FACS Calibur flow cytometer (Becton Dickson, Milan, Italy) using Cell Quest software [22]. Mean fluorescent intensity was compared with control staining using an irrelevant isotype-matched mouse monoclonal antibody.

### Immunofluorescence microscopy

BEAS-2B cells were cultured on 8-well glass Labtek slides (Nalge Nunc International) under different experimental conditions. The cells were treated with 100 μg/ml pidotimod or 10 ng/ml TNF-α used alone or in association, for 24 h. BEAS-2B cells were fixed in ice-cold methanol for 5 min. After blocking BEAS-2B were labelled with mouse anti TLR-2 Abs (TL2.1; Santa Cruz Biotechnology), then with a goat anti-mouse antibody (Alexa Fluor488; Invitrogen).

### IL-8 assay

BEAS-2B cells were incubated for 24 h with 10 ng/ml TNF-α, 50 μg/ml zymosan or pidotimod (10, 100 μg/ml), in the absence or presence of TNF-α and zymosan. After the treatments the supernatant were collected and IL-8 levels were quantified by enzyme-linked immunosorbent assay (ELISA) kits (R&D Systems, Minneapolis, USA), according to the manufacturer’s instructions.

### Preparation of cytoplasmic extracts for TLR-2 analysis and activated extracellular-signal-regulated kinase (ERK)1/2 analysis

For TLR-2 analysis, BEAS-2B cells were grown to 90% confluence in 24 well plates and treated with pidotimod (10, 100 μg/ml), 10 ng/ml TNF-α, 50 μg/ml zymosan, used alone or in association, for 24 h. For ERK1/2 analysis, cells were equally plated and cultured until 90% confluence and then exposed to pidotimod, TNF-α or TNF-α with pidotimod, for 5 to 60 min [23]. After the treatments the cells were scraped and lysed with lysis buffer [20 mM Tris–HCl buffer (pH 7.4)], containing 150 mM NaCl, 1 mM EDTA, 1nM EGTA, 1 mM sodium orthovanadate, 1% (v/v) Triton X-100, 1 mM PMSF, 10 μg/ml leupeptin, and 10 μg/ml aprotinin) for 5 min on ice. The lysates were then centrifuged at 12,000 g for 5 min [23].

### Preparation of cytoplasmic and nuclear extracts for NF-kB analysis

BEAS-2B cells were grow to 90% confluence in 6 well plates and treated with 100 μg/ml pidotimod, TNF-α or TNF-α with pidotimod for 1 h. Extracts were prepared using a method described by Leslie J. Crofford [24]. Briefly, cells resuspended in cytoplasmic buffer (10 mM HEPES, pH 7.9, 10 mM KC1, 0.1 mM EDTA, 0.1 mM EGTA, 1 mM dithiothreitol [DTT], and protease inhibitors) on ice for 15 minutes, after which Triton X-100 was added to a final concentration of 0.25%. Intact nuclei were pelleted by centrifugation, and the cytoplasmic extract was immediately frozen. Nuclei were resuspended in high-salt buffer (20 mM HEPES, pH 7.9, 400 mM NaCI, 1 mM EDTA, 1 mM EGTA, 1 mM DTT, and protease inhibitors) for 15 min at 4°C. The nuclear extract was collected after centrifugation at 13,500 g for 5 min at 4°C.

### Western blot analysis

The protein concentration of the lysates was measured using the Bio-Rad protein assay. Equal amounts of protein were resolved with 10% or 12% SDS-PAGE and transferred to PVDF membranes. Membranes were blotted for TLR-2 and p65 NF-kB (Santa Cruz Biotechnology), total ERK1/2 and phosphorylated ERK1/2 (Cell Signaling, Technology, Beverly, MA, USA), GAPDH (Novus Biological, Inc, Segrate, Milan, Italy), Lamin A/C (Santa Cruz Biotechnology Inc, Segrate, Milan, Italy) [23]. After incubation with HPR-conjugated anti-rabbit o anti-mouse antibody (Cell Signaling Technology), the bands were detected using enhanced chemoluminescence (ECL, Pierce, Celbio, Italy) and relevant band intensities were quantified using a Versadoc Imaging System model 3000 (Biorad Laboratories, Hercules, CA, USA).

### Statistical analysis

Statistical evaluation was performed using the statistical software package GraphPad Prism 3.02 (GraphPad Software, San Diego, CA, USA). The results were expressed as mean ± standard error of the mean (SEM) and t test or the Mann–Whitney test were used. Probability values (P < 0.05) were considered as statistically significant.

## Results

### Airway epithelial cell viability

Assessment of BEAS-2B cells by trypan blue dye exclusion test showed that after 24 h incubation, cell viability > 90% and was similar in cultures containing pidotimod (10 e 100 μg/ml), zymosan, TNF-α or pidotimod plus zymosan or TNF-α (not shown). Similar results were obtained exposing BEAS-2B cells for 48 h (not shown).

### ICAM-1 expression by airway epithelial cells

**Figure 1:**
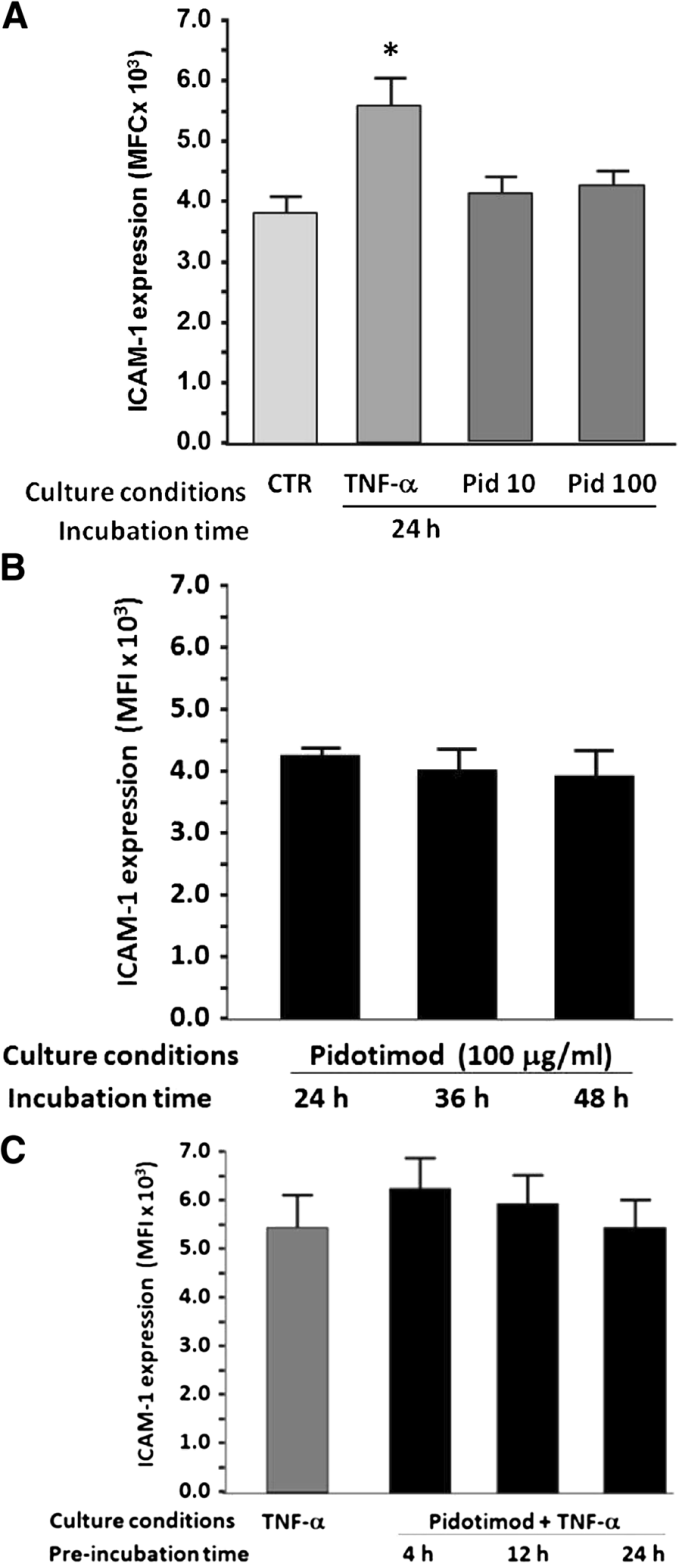
**ICAM 1 expression in BEAS-2B cells. **Flow cytometric analysis of ICAM-1 expression on cells: **A**) untreated (CTR), treated with TNF-α or pidotimod (pid 10 and 100 μg/ml) for 24 h; **B**) treated with 100 μg/ml pidotimod for different time periods (24, 36 and 48 h); **C**) pre-treated with pidotimod for different time (4, 12 and 24 h) and then stimulated with TNF-α for additional 24 h. ICAM-1 expression is quantified as mean fluorescence intensity (MFI) on the ordinate and the different culture conditions are shown on the abscissa. The data are expressed as mean ± SEM. *p < 0.05 versus control. The results shown are representative of three independent experiments.

To investigate the effect of pidotimod on ICAM-1 expression, BEAS-2B cells were exposed to pidotimod (10, 100 μg/ml), TNF-α (10 ng/ml) or pre-treated with pidotimod for different time (4, 12 or 24 h) and then stimulated with TNF-α for additional 24 h. The cells were stained with antibody anti-human ICAM-1. Result obtained by flow cytometric analysis showed that the constitutive expression of ICAM-1 by BEAS-2B cells was significant up-regulated by 24 h exposure of the cells to TNF-α (p < 0.05), but not to pidotimod (Figure 1A). No changes in ICAM-1 expression were obtained exposing the cells to pidotimod for 36 h or 48 h (Figure 1B) and no modifications in the TNF-α -induced increase in ICAM-1 expression was observed pre-treating the cells with pidotimod for 4, 12 or 24 h (Figure 1C).

### TLR-2 expression by airway epithelial cells

**Figure 2:**
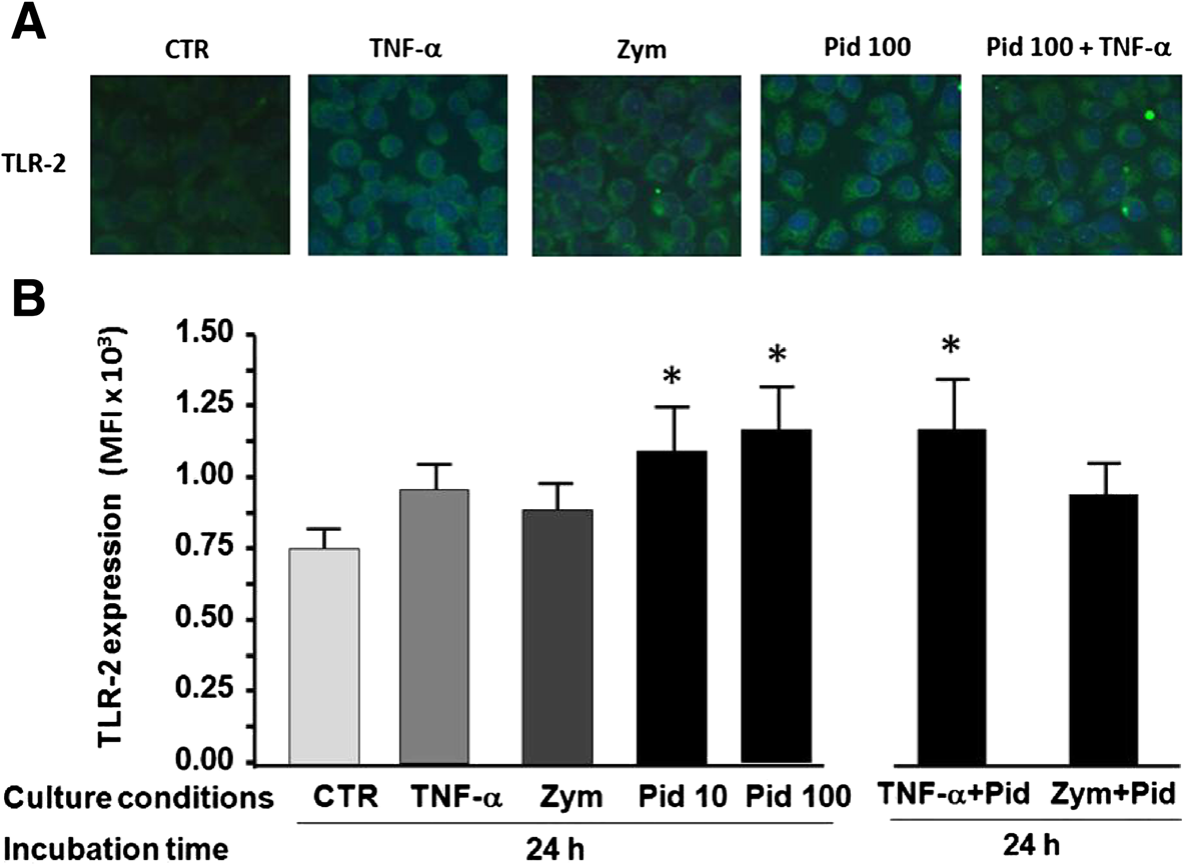
**Immunofluorescence and flow cytometry analysis of TLR-2 expression induced by pidotimod. **(**A**) Representative images of TLR-2 expression collected by fluorescence microscopy in cell cultures untreated (CTR) or treated with TNF-α, zymosan, pidotimod (Pid 100 μg/ml) or pidotimod (Pid 100 μg/ml) plus TNF-α for 24 h; **B**) Histograms displaying the flow cytometric analysis of TLR-2 expression on cells treated with TNF-α, zymosan, pidotimod (Pid 10, 100 μg/ml), or pidotimod (Pid 100 μg/ml) plus TNF-α (TNF-α Pid or zymosan (Zym + Pid, for 24h. TLR-2 expression is quantified as mean fluorescence intensity (MFI) on the ordinate and the different culture conditions are shown on the abscissa. The data are expressed as mean ± SEM.*p < 0.05 versus control. The results shown are representative of three independent experiments.

**Figure 3:**
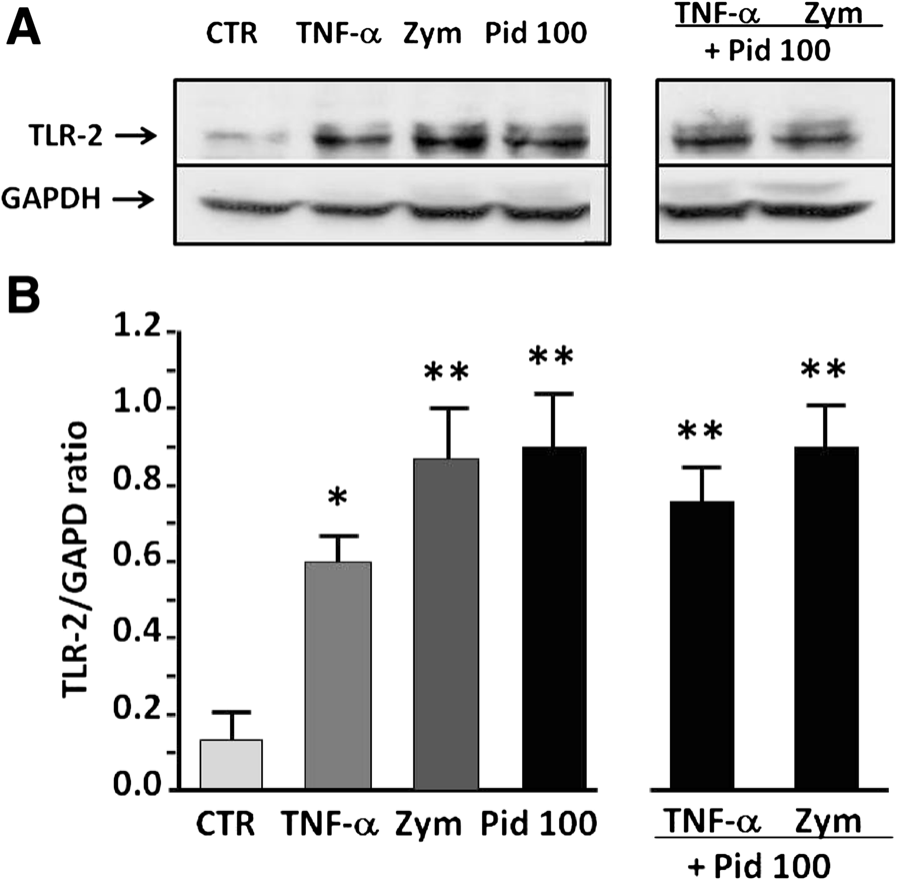
**Western blot analysis of TLR-2 expression induced by pidotimod. **(**A**) Representative western blot of TLR-2 and GAPDH expression of BEAS-2B incubated with medium alone (CTR) or treated with TNF-α, zymosan (Zym), pidotimod (Pid 100 μg/ml) or pidotimod (Pid 100 μg/ml) plus TNF-α or zymosan (Zym), for 24 h.(**B**) The densitometric analysis of TLR-2 expression, normalized to GAPDH, is shown on the ordinate and the different culture conditions on the abscissa. The data are expressed as mean ± SEM. *p < 0.05 and **p < 0.01 versus control. The results shown are representative of three independent experiments.

To analyze the expression of TLR-2, BEAS-2B cells were exposed for 24 h to pidotimod (10 and 100 μg/ml), TNF-α, zymosan or pidotimod with TNF-α or zymosan. The enhanced TLR-2 expression induced by exposure to the stimuli detected by immunofluorescence microscopy (Figure 2A) was then quantified by flow cytometric analysis and found to be significant only after exposed to pidotimod 10 or 100 μg/ml (p < 0.05) and the association pidotimod 100 + TNF-α (p < 0.05) (Figure 2B). Western blot (Figure 3A) and densitometric analysis demonstrated that, at protein level, the effect was statistically significant also for TNF-α (p < 0.05), zymosan (p < 0.01) and for the combinations zymosan with pidotimod (p < 0.01) (Figure 3B). Pre-incubation of the cells with pidotimod for 4, 12 or 24 h did not modify the results (not shown).

### IL-8 release

**Figure 4:**
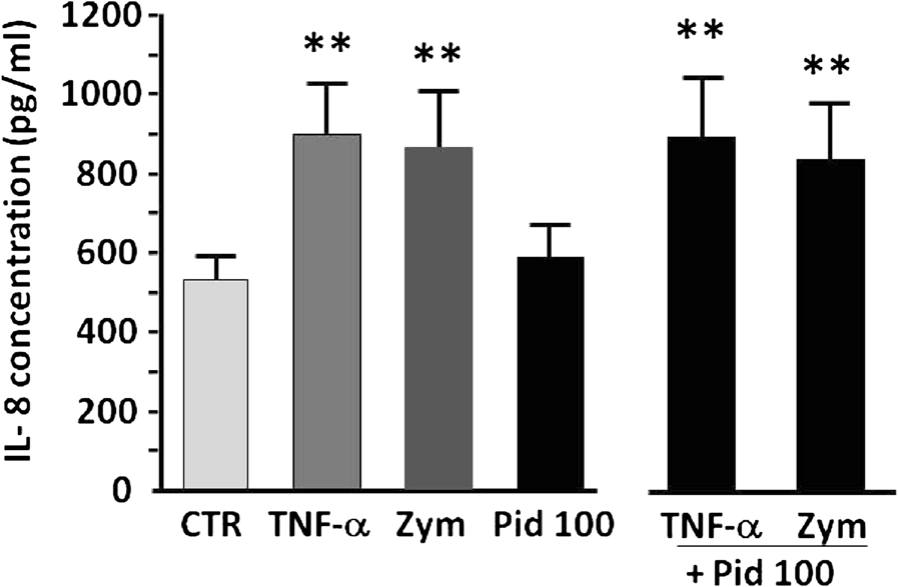
**Assessment of IL-8 secretion. **IL-8 concentration in the supernatants of BEAS-2B incubated with medium alone (CTR) or treated with TNF-α zymosan (Zym), pidotimod (Pid 100 μg/ml), TNF-α and pidotimod (TNF-α, Pid or zymosan and pidotimod (Zym + Pid for 24 h, evaluated by ELISA. IL-8 are expressed as mean ± SEM. **p < 0.01 versus control.

BEAS-2B cells were exposed for 24 h to pidotimod (100 μg/ml), TNF-α, zymosan or TNF-α and zymosan with pidotimod. The detectable IL-8 concentrations found in the supernatants of cell cultured in medium alone, were significantly increased in cell culture exposed for 24 h to TNF-α or zymosan (p < 0.01, each comparison), but not to pidotimod (Figure 4). No further modifications in the TNF-α - or zymosan-induced increase in IL-8 release was observed with the addition of pidotimod to the cell cultures. Finally, preincubation of the cells with pidotimod did not modify the results (not shown).

### ERK1/2 pathway activation

**Figure 5:**
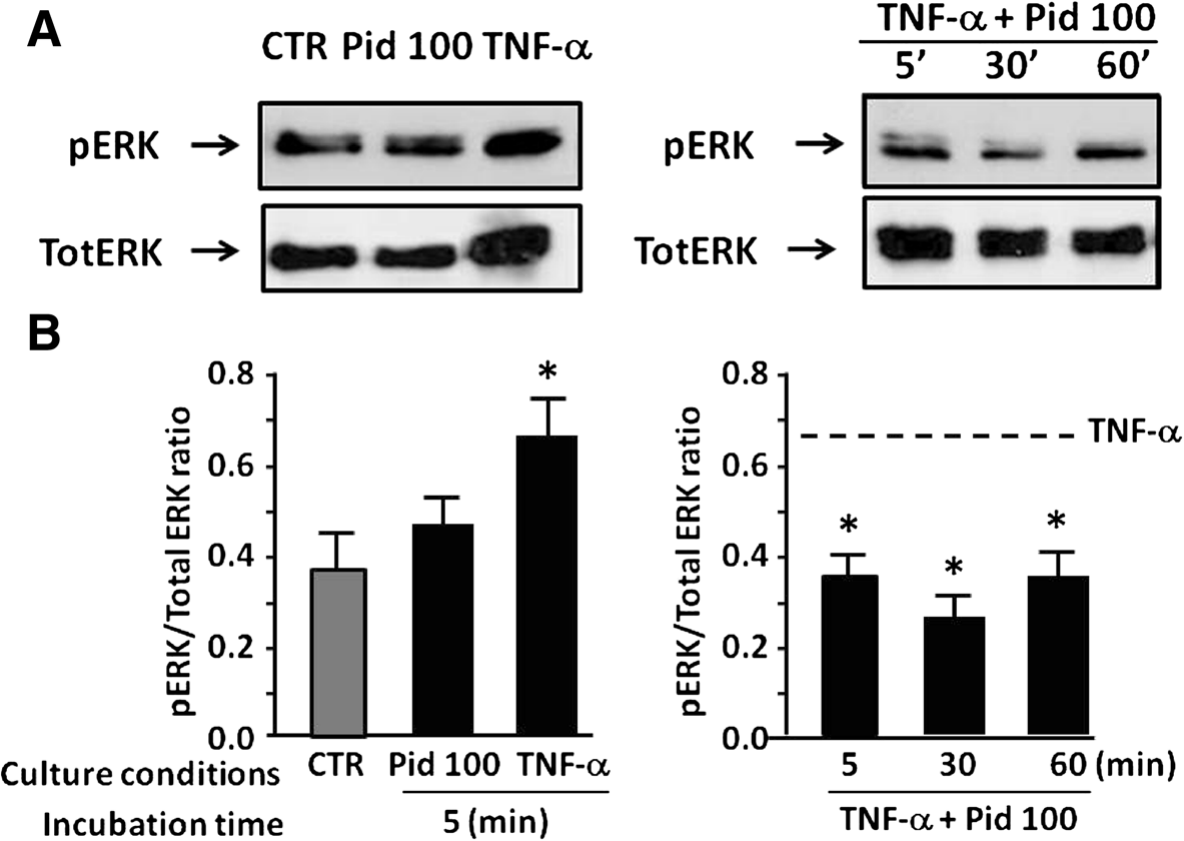
**Detection by Western blot of ERK1/2 phosphorylation. **(**A**) Representative western blot of phosphorylated and total ERK1/2 of BEAS-2B cells untreated (CTR) or exposed to TNF-α or pidotimod (Pid) for 5 min or TNF-α with pidotimod (TNF-α + Pid) for 5 to 60 min. (**B**) The densitometric analysis of ERK1/2 phosphorylation, normalized to total ERK1/2 is shown on the ordinate and the different culture conditions on the abscissa. The data are expressed as mean ± SEM.*p < 0.05 versus control. The results shown are representative of three independent experiments.

For ERK1/2 analysis, confluent BEAS-2B cells were exposed to pidotimod, TNF-α or TNF-α with pidotimod for 5 to 60 min. An increase threonine/tyrosine phosphorylation of ERK1/2 was already detectable by Western blotting at 5 min in cell exposed to TNF-α, but not to pidotimod (100 μg/ml) (Figure 5A). In contrast, the addition of pidotimod (100 μg/ml) to the cells exposed to TNF-α induced a detectable decrease in ERK1/2 phosphorylation (Figure 5A). Densitometric analysis demonstrated that the inhibitory effect was complete at 5, 30 and 60 min (Figure 5B).

### NF-kB expression and translocation

**Figure 6:**
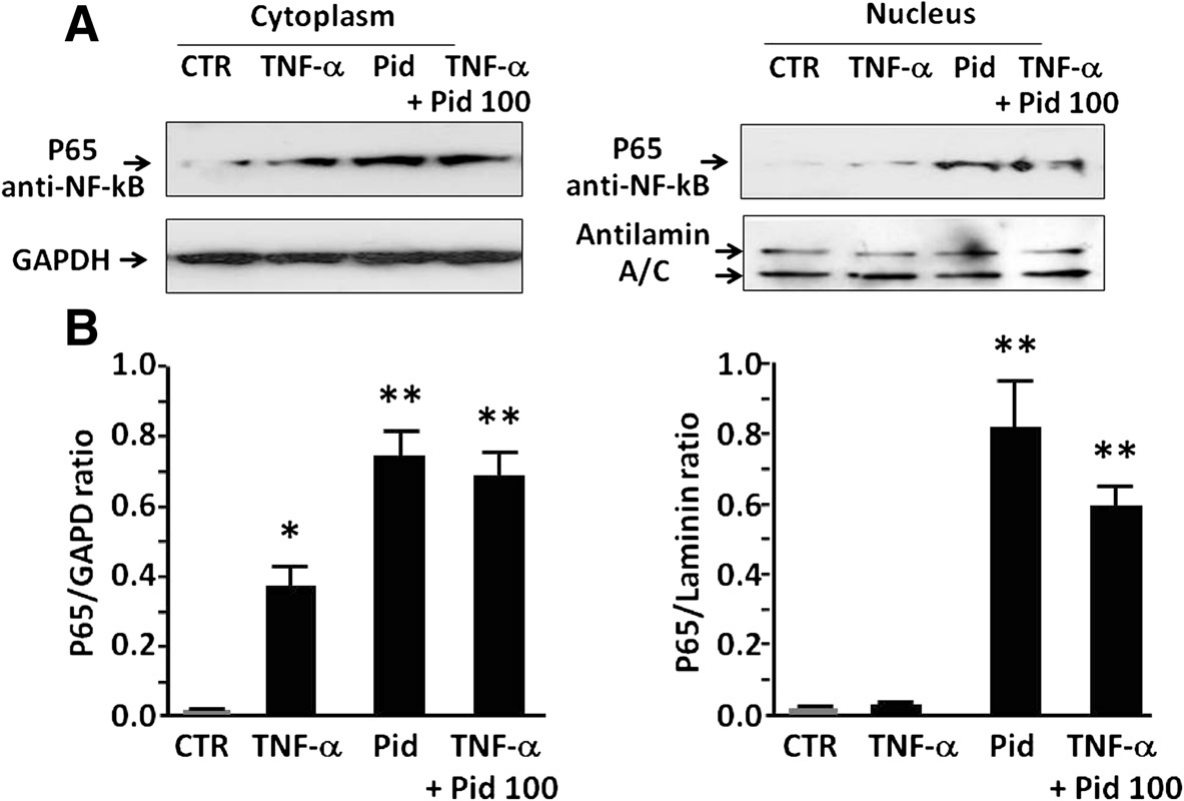
**Detection by Western blot of NF-kB expression. **(**A**) Representative western blots for NF-kB p65 levels in the cytoplasm and in the nuclear extracts of BEAS-2B cultured with medium alone (CTR) or exposed to TNF-α, 100 μg/ml pidotimod (Pid 100), or TNF-α and pidotimod (TNF-α + Pid 100) for 24 h. GAPDH and Laminin A/C were used as housekeeping protein. (**B**) The densitometric analysis of NF-kB data, normalized to GAPDH (cytoplasm) or laminin A/C (nucleus), are shown on the ordinate and the different culture conditions on the abscissa. The data are expressed as mean ± SEM. *p < 0.05 versus control. The results shown are representative of three independent experiments.

BEAS-2B cells were treated with TNF-α, 100 μg/ml pidotimod or TNF-α with pidotimod for 1h. NF-kB was detected by western blot analysis of cytoplasmic and nuclear extracts. NF-kB p-65 expression was upregulated in the cytoplasmic compartment after exposure to TNF-α or pidotimod and associated with NF-kB nuclear translocation (Figure 6A and B). Similar results were obtained in the experiments performed with pidotimod 10 μg/ml (not shown).

## Discussion

Using the BEAS-2B human bronchial epithelial cells line we have shown that pidotimod is able to induce in vitro cellular changes potentially useful in enhancing the capability of the host to fight respiratory infections. We found that exposure of BEAS-2B cells to pidotimod had no effect on ICAM-1 expression and IL-8 release, while a detectable upregulation of TLR-2 expression was observed by fluorescence microscopy, cytofluorimetry and western blot analysis. Pidotimod was also effective in inducing a remarkable inhibition of TNF-α-induced ERK1/2 phosphorylation and, at the opposite, a significant increase in NF-kB protein expression and NF-kB nuclear translocation.

Pidotimod is a synthetic dipeptide molecule with biological and immunological activity on both the adaptive and the innate immune responses [25]. In vitro studies, both from animal and human specimens, have demonstrated a significant activity on both the innate and the adaptive immune responses, explaining the remarkable results of the clinical studies, where reduction in the rate of recurrent infections of the upper respiratory tract has been observed [25-27].

Emerging evidence from epidemiologic, clinical and animal studies indicates that viral infections, the leading cause of respiratory morbidity in young children, is an important environmental stimulus for airway injury and remodeling, resulting in bronchial hyperreactivity, impaired lung function and, potentially, persistent asthma [28]. The availability of PCR techniques has lead to a significant improvement in virus detection rate and shown that HRVs, principally known as the “common cold” viruses, are involved in the pathogenesis of wheezing disorders in 41-45% of young children [29]. Approximately 90% of the different HRV serotypes known utilize, as specific receptor ICAM-1, a molecule expressed on the surface of airway epithelial cells [30]. HRV infection upregulates ICAM-1 expression on airway epithelial cells, thus facilitating further viral attachment and entry [31]. Besides being a major receptor for HRVs, ICAM-1 is also involved in the transmigration across airway epithelial monolayers of neutrophils and their activation [32], and in disorders characterized by neutrophil-mediated acute lung injury [33]. Therefore the observation that in our experimental model pidotimod did not increase the constitutive or the TNF-α -induced ICAM-1 expression may be interpreted as a “protective function”, to avoid enhanced susceptibility to human HRV and neutrophil-mediated damage to the airway surface. A similar interpretation can be made for the results on IL-8 release.

IL-8 is a powerful chemotactic and paracrine mediator for neutrophils, and infiltration of activated neutrophils play a key role in pulmonary inflammation and oxidative injury [34], a characteristic feature of respiratory viral infections [35,36].

On the contrary, pidotimod enhanced the constitutive TLR-2 expression, raising questions on the activity of pidotimod on airway epithelial cells. The expression of surface molecules (such as ICAM-1 and TLR-2) is regulated not only by different stimuli [31,37,38] but also by dissimilar intracellular pathways that may be triggered by the same stimulus [38-41]. An example is the activity of IFN-γ, able to have opposite effects on ICAM-1 and TLR-2 expression (up- and down-regulation, respectively) on different cell types [37,38,42]. The characteristics of pidotimod, to be able to regulate differently the expression of surface molecules has already been demonstrated by Gourgiotis D. and co-workers [43]. Using blood mononuclear cells, isolated from atopic asthmatic and normal children, they showed that through unknown intracellular mechanisms pidotimod downregulated the expression of CD30 induced by phytohaemoagglutinin but had no effect on human leukocyte antigen (HLA)-DR molecules [43].

TLRs are a class of proteins that play a key role in the innate immune system, recognizing structurally conserved molecules derived from microbes [44]. TLRs are a type of pattern recognition receptor and recognize molecules that are broadly shared by pathogens but distinguishable from host molecules, collectively referred to pathogen-associated molecular patterns (PAMPs). Of the ten mammalian TLR proteins identified thus far, TLR-2 seems to be the least discriminating, since it recognizes many bacterial, fungal, viral, and certain endogenous substances [44,45]. Recognition of PAMPs results in internalization and phagocytosis of bound molecules and in cellular activation with release of cytokines, chemokines and various interleukins [44,45]. Cytokines participating in this “nonspecific” immune defense include various interleukins, such as TNF-α, IL-1α, IL-1β, IL-6, IL-8 and IL-12. PAMPs-TLR interactions on the surface of the cells are sufficient to activate the Ca2^+^ fluxes associated with induction of NF-kB in the airway [46]. Recent studies have implicated TLR, and especially TLR-2 and TLR-4, as sentinel receptors able to signaling the interaction of the host cells with bacterial pathogens via an NF-kB-mediated pathway [47]. Interestingly, using murine macrophages it was shown that TLR-2 mRNA may be induced after bacterial infection with the involvement of several cytokines, including IL-1 α, and GM-CSF, but that NF-kB is necessary for maximal TLR-2 transcription [48], further underlining the tight connections between NF-kB and TLR-2.

The different effects of pidotimod on BEAS-2B cells here reported may be at least partially explained by the opposite effects on some cell functions produced by ERK1/2 phosphorylation or NF-kB activation.

Extracellular-signal-regulated kinases (ERKs) or classical mitogen-activated protein (MAP) kinases are widely expressed protein kinase intracellular signalling molecules that are engaged in the regulation of meiosis, mitosis, but also in several post mitotic functions, including activities involved in inflammatory and defense processes [49,50]. These include opposite effects on ICAM-1 and TLR-2 expression. In agreement with our results on BEAS-2B cells, stimulation of ERK1/2 phosphorylation by cysteinyl leukotriene D4 resulted in an increased ICAM-1 expression by 16HBE bronchial epithelial cells, associated with enhanced eosinophil adhesion [51]. In contrast, inhibition of ERK1/2 phosphorylation [52] by up-regulation of MAP kinase phosphatase-1 by glucocorticoids increased TLR-2 expression by HeLa epithelial cells.

NF-kB is a protein complex, found in almost all animal cell types, that controls DNA transcription and is involved in cellular responses to a variety of stimuli such including bacterial or viral antigens [53,54]. NF-kB plays a key role in regulating a quick immune response to infections because it belongs to the category of “rapid-acting” primary transcription factors, i.e. present in cells in an inactive state and not requiring new protein synthesis to be activated and is involved in the regulation of reactive oxygen species, cytokines and adhesion molecules production [54-57].

The identification of TLRs as specific pattern recognition molecules and the finding that, not only their expression is regulated by NF-kB [42], but also that TLR stimulation leads to NF-kB activation has improved our understanding on both the innate and the adaptive immune responses to infections.

Surprisingly, NF-kB activation induced by pidotimod did not result in an increase in IL-8 release. However, in human airway epithelial cell cultures and in mice inhibition of ERK1/2 phosphorylation suppress IL-8 production induced by exposure to sub toxic doses of cadmium [58]. Moreover, other transcriptional factors are involved in IL-8 production and release, including activator protein 1 (AP-1), and signal transducer and activator of transcription proteins (STAT) [59,60]. Rather than pidotimod, elevated intracellular levels of reactive oxygen species (ROS) in lung epithelial cells can activate these pathways [60,61]. Therefore, upstream of these transcriptional factors, ROS-sensitive signaling pathways, including mitogen-activated protein kinases and protein kinase C, may be involved in the increase in inflammatory gene activation [56,57].

## Conclusion

We have shown that pidotimod, a synthetic dipeptide used in the prevention of recurrent respiratory infection in children, is able to modulate airway epithelial cells functions involved in host-virus possibly through NF-kB activation. The obvious limitation of the study is that the experiments here reported were obtained in a human cell line. If confirmed in vivo, these activities may, at least in part, clarify the mechanism of action of this molecule at airway level.

## Abbreviations

HRV: Human rhinovirus; RIS: Respiratory insufficiency syndrome; TNF-α: Tumor necrosis factor-α; RANTES: Regulated by activation normal T cell expressed and secreted; ICAM-1: Intercellular adhesion molecule-1; TLR-2: Toll-like receptor-2; IL: Interleukin; NF-kB: Nuclear factor-kappa B; ERK: Extracellular-signal-regulated kinase; ELISA: Enzyme-linked immunosorbent assay; DTT: Dithiothreitol; IFN-y: Interferon- y; HLA-DR: Human leukocyte antigen-DR; PAMP: Pathogen-associated molecular pattern; MAP kinase: Mitogen-activated protein kinase; AP: Activator protein; STAT: Signal transducer and activator of transcription proteins; ROS: Reactive oxygen species.

## Competing interests

GAR. Received in the past five years reimbursements, fees and funding from Polichem S.A., Luxembourg and Valeas S.p.A., Milan, Italy, that way gain or lose financially from the publication of this manuscript.

SC and MS do not declare any-financial competing interests.

## Authors’ contributions

All the authors have made substantial contributions to conception and design of the study, to the analysis, interpretation and acquisition of data of the data. SC performed all the in vitro experiments and MS the statistical analysis. 2) All the authors have been involved in drafting the manuscript and revising it critically for important intellectual content. All authors read and approved the final manuscript.
